# The Role of Registered Dietitians in Cancer Palliative Care: Responsibilities, Challenges, and Interdisciplinary Collaboration—A Cross-Sectional Survey

**DOI:** 10.3390/curroncol32050275

**Published:** 2025-05-12

**Authors:** Saori Koshimoto, Koji Amano, Naoharu Mori, Atsuko Imai, Manami Sasaki, Miho Miyajima, Takashi Takeuchi

**Affiliations:** 1 Liaison Psychiatry and Psycho-Oncology Unit, Department of Psychiatry and Behavioral Sciences, Graduate School of Medical and Dental Sciences, Institute of Science Tokyo, 1-5-45 Yushima, Bunkyo-ku, Tokyo 113-8519, Japan; miholppm@tmd.ac.jp (M.M.); okaspsyc@tmd.ac.jp (T.T.); 2 Department of Health and Nutritional Science, Tokoha University, 1230 Miyakoda-cho, Hamana-ku, Hamamatsu 431-2101, Japan; 3 Department of Supportive and Palliative Care, Osaka International Cancer Institute, 3-1-69 Otemae, Chuo-ku, Osaka 541-8567, Japan; kojiamano@oici.jp; 4 Department of Palliative and Supportive Medicine, Graduate School of Medicine, Aichi Medical University, 1-1 Yazakokarimata, Nagakute 480-1195, Japan; nmori@aichi-med-u.ac.jp; 5 Department of Nutritional Management, Sagami Women’s University, 2-1-1 Bunkyo, Minami-ku, Sagamihara 252-0383, Japan; imai_atsuko@isc.sagami-wu.ac.jp; 6 Department of Palliative Medicine, Tohoku University Hospital, 1-1 Seiryomachi, Aoba-ku, Sendai 980-8574, Japan

**Keywords:** palliative care, cancer patients, registered dietitian, interdisciplinary collaboration, ward round, challenges encountered

## Abstract

Registered dietitians (RDs) in palliative care help maintain patients’ quality of life by providing personalized nutritional support that alleviates eating-related distress. This study aimed to clarify the role of RDs in palliative care by examining their responsibilities and challenges in caring for cancer patients. A nationwide mailed survey was conducted in 2022, focusing on RDs involved in cancer palliative care. One RD per facility was included from all 501 hospitals accredited by Japan’s Ministry of Health, Labour and Welfare. Multivariate analysis identified factors related to collaboration with palliative care teams and challenges in cancer care. Responses from 325 RDs (63.9%) across 325 hospitals (63.9%) were analyzed. Among RDs who consistently collaborated with the palliative care team (PCT), significant associations (*p* < 0.05) were found with exclusive engagement in cancer/palliative care, providing nutritional counseling to inpatients, the frequency of ward rounds, and individualized meal provision. Challenges included the following: “I struggled with determining appropriate food choices for patients unable to eat”, and “Metabolic complications like cachexia hindered my ability to provide adequate support”. RDs play a crucial role in providing individualized meals for cancer patients through PCT collaboration and ward rounds. To ensure effective support in challenging situations, RDs must be exclusively engaged in palliative care and receive specialized education.

## 1. Introduction

Palliative care is a holistic medical approach that addresses the physical symptoms of cancer patients as well as their psychiatric and psychological distress through mental health care [1]. A comprehensive approach to physical, psychosocial, and spiritual issues is necessary at diagnosis, throughout treatment, and in the home setting to ensure that patients and their families receive the highest possible quality of care and recuperation [2].

Common interventions include nutritional support [3]. Malnutrition and cachexia contribute to delayed postoperative recovery, reduced therapeutic efficacy, increased infection risk, prolonged hospitalization, decreased activities of daily living, and diminished quality of life (QOL) [4,5,6,7,8]. Therefore, intervention by registered dietitians (RDs) is important [9,10,11]. A survey of cancer outpatients reported that low QOL and eating-related distress are key factors influencing the decision to seek nutritional counseling [3]. Notably, patients with cancer cachexia and their families express concerns about eating-related distress [12]. They often experience difficulties with food and nutrition and require comprehensive support that addresses psychological aspects such as anxiety and depression [13,14,15]. RDs play a vital role in palliative care, as nutritional support must address both physical needs and psychosocial aspects [9]. Within the healthcare system, RDs are expected to collaborate with palliative care teams (PCTs) and provide nutritional management tailored to patients’ symptoms and preferences [16]. However, the required competencies for RDs in palliative care remain unstandardized, and interdisciplinary collaboration in cancer cachexia management and eating-related distress remains inadequate. We performed a questionnaire survey to explore the responsibilities of RDs in palliative care, the challenges they encounter in patient care, and their role in improving overall patient support.

## 2. Materials and Methods

### 2.1. Subjects

Study participants were RDs engaged in cancer palliative care or cancer care who completed a self-administered, anonymous questionnaire survey. In 2022, nationwide questionnaires were mailed to RDs at 509 hospitals that met the criteria for palliative care facilities in Japan and were listed in the institutional directory of the Ministry of Health, Labour and Welfare. One representative RD from each hospital was requested to respond, and completed questionnaires were requested to be returned in a stamped, self-addressed envelope within one month.

### 2.2. Questionnaire

The demographic data collected included participants’ age, sex, area of practice, years of experience, nature of practice, and prior educational experience related to palliative and end-of-life care. Based on a review of previous studies on palliative care and nutrition, we identified 10 key questions that address the challenges faced by RDs in the care of palliative and terminally ill patients. The questionnaire included the following items:

➢ I struggled with determining appropriate food choices for patients unable to eat.
➢ Metabolic complications, such as cachexia, hindered my ability to provide adequate nutritional support.
➢ I was unable to meet patients’ dietary wishes.
➢ I was concerned that eating might cause severe complications such as vomiting or choking.
➢ The patient refused to eat.
➢ I was consulted on decisions regarding the continuation or discontinuation of tube feeding, gastrostomy, or intravenous nutrition.
➢ Providing psychological support to the patient was challenging.
➢ Differences in opinions among staff members arose due to varying perspectives on patient care.
➢ I felt that my overall knowledge and experience in palliative care were insufficient.
➢ Providing support to patients’ families was challenging.

These items were developed independently based on previous studies [3,9,12,13,14]. Facility-related data included the number of beds, meal management system, availability of individualized meals, and frequency of ward rounds.

### 2.3. Statistical Analysis

The level of collaboration with the PCT was compared between two groups: (1) RDs who consistently collaborated as team members or maintained continuous involvement with the team (consistently collaborated group [CC group]) and (2) RDs who collaborated with the team on an as-needed basis (collaborated as-needed group [AN group]). Comparisons between the PCT and the CC and AN groups were performed using Fisher’s exact test for nominal variables and the Mann–Whitney U test for ordinal variables. To identify factors associated with collaboration between RDs and PCT, a multivariate analysis was performed using the following independent variables: certification; responsibilities; nutritional counseling responsibilities; ward rounds; provision of meals; level of individualized meal provision; and education in palliative care. In addition, in order to identify the factors influencing the challenging situations encountered by RDs, challenging situations were used as dependent variables for two groups: (1) “often/sometimes” and (2) “not often/never”. Independent variables included years of experience in cancer care; certification as an RD specializing in cancer; exclusively engaged in palliative care or cancer care; meal rounds; fully individualized meals based on patient preferences; level of collaboration with the palliative care team; and pre-graduate education and post-graduate education on palliative care. Multiple logistic regression was used for multivariate analysis. Covariate correlations were assessed to exclude multicollinearity prior to logistic regression analysis. All statistical analyses were performed using SPSS (IBM Corp. Released 2023. IBM SPSS Statistics for Windows, Version 29.0.2.0 Armonk, NY, USA: IBM Corp.). A two-tailed test was applied, and statistical significance was set at *p* < 0.05.

## 3. Results

Of the 509 palliative care facilities, responses were received from 329 RDs (response rate of 64.6%). After excluding responses that did not provide consent, 325 facilities (63.9%) and 325 RDs (63.9%) were included in the final analysis. Table 1 shows the characteristics of the RDs. Table 2 describes the characteristics of their respective facilities.

Table 3 shows the association between the role of RDs and their collaboration with the PCT. Compared with the AN group (25.8%, n = 84), the CC group (74.2%, n = 241) was significantly more likely to be dedicated to cancer/palliative care (*p* = 0.003), responsible for nutritional counseling for hospitalized patients (*p* = 0.005), participate in ward rounds more frequently (*p* < 0.001), provide individualized meals (*p* = 0.004), and ensure the availability of completely individualized meals based on patients’ wishes (*p* = 0.035).

Multivariate analysis identified three significant factors influencing consistent collaboration with the PCT: exclusively engaged in palliative care or cancer care (OR = 3.18; 95% CI: 1.27–7.98; *p* = 0.014); the frequency of ward rounds, with daily (OR = 9.70; 95% CI: 2.23–42.12; *p* = 0.002) and several times per week (OR = 9.35; 95% CI: 2.19–39.97; *p* = 0.003) being significant predictors; and the type of meal provision, with the implementation of individualized meals (OR = 3.50; 95% CI: 1.01–12.07; *p* = 0.048) also being a significant predictor (Table 4).

**Figure 1:**
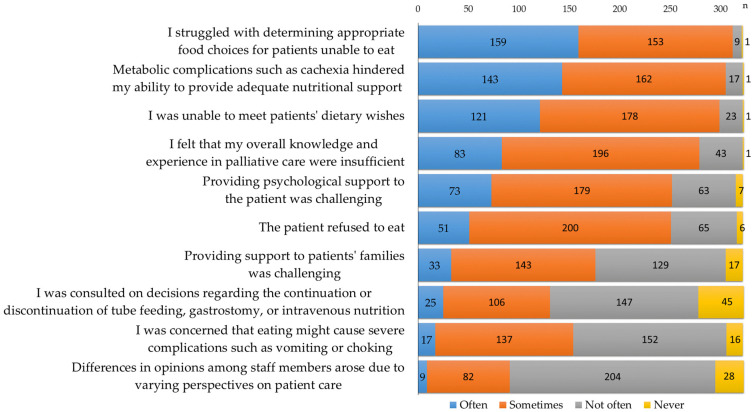
Challenges encountered by RDs in providing care for palliative patients (n = 325).

Figure 1 shows the challenges encountered by RDs in providing care for palliative patients. More than 90% of RDs reported “often” or “sometimes” experiencing difficulties, including “I struggled with determining appropriate food choices for patients unable to eat”, “Metabolic complications such as cachexia hindered my ability to provide adequate support”, and “I was unable to meet patients’ dietary wishes”.

To identify the factors influencing these challenges, multivariate analysis was performed using the following independent variables: years of clinical experience, certification as a registered dietitian specializing in cancer (reference: no certification), exclusively engaged in palliative care or cancer care (reference: not exclusively engaged), meal round responsibilities (reference: no meal rounds), fully individualized meals based on patient preferences (reference: no fully individualized meals), level of collaboration with the PCT, pre-graduate education (reference: no pre-graduate education), and post-graduate education (reference: no post-graduate education).

The provision of fully individualized meals was significantly associated with a decrease in the number of challenging situations for RDs, such as “I struggled with determining appropriate food choices for patients unable to eat” (OR = 0.22; 95% CI: 0.05–0.88; *p* = 0.032) and “Metabolic complications, such as cachexia, hindered my ability to provide adequate nutritional support” (OR = 0.30; 95% CI: 0.11–0.85; *p* = 0.023). The provision of fully individualized meals based on patient preferences (OR = 0.35; 95% CI: 0.15–0.86; *p* = 0.022) and pre-graduate education (OR = 0.22; 95% CI: 0.08–0.60; *p* = 0.003) were significantly associated with fewer challenging situations in which RDs reported, “I was unable to meet patients’ dietary wishes”. Certification as a registered dietitian specializing in cancer (OR = 0.39; 95% CI: 0.18–0.84; *p* = 0.016) and participation in meal rounds (OR = 0.47; 95% CI: 0.22–0.98; *p* = 0.044) were significantly associated with fewer challenging situations in which RDs reported, “I felt that my overall knowledge and experience in palliative care were insufficient”. Years of experience in cancer care (OR = 0.95; 95% CI: 0.91–1.00; *p* = 0.037) and pre-graduate education (OR = 0.46; 95% CI: 0.22–0.99; *p* = 0.048) were significantly associated with fewer challenging situations in which RDs stated, “The patient refused to eat”. However, post-graduate education was significantly associated with an increase in challenging situations, such as “Providing support to patients’ families was challenging” (OR = 2.01; 95% CI: 1.15–3.52; *p* = 0.015). Additionally, participation in meal rounds was associated with an increased occurrence of “I was consulted on decisions regarding the continuation or discontinuation of tube feeding, gastrostomy, or intravenous nutrition” (OR = 2.30; 95% CI: 1.40–3.79; *p* = 0.001) (Table 5).

## 4. Discussion

### 4.1. Collaboration with the Palliative Care Team

RDs play a crucial role in providing nutritional support to cancer patients, such as addressing their dietary preferences and eating-related distress, in palliative care settings [17,18]. This study aimed to examine the responsibilities of RDs involved in palliative care, identify the challenges they encounter when working with patients, and explore the interdisciplinary collaboration systems associated with the role of RDs.

A comparison between the CC group and the AN group revealed that the presence of a dedicated RD specializing in palliative cancer care significantly enhances collaboration with the PCT. However, the proportion of dedicated RDs remains low (22%), indicating a need for more specialized RDs to provide tailored support for patients’ dietary preferences and eating-related distress [19,20,21]. Our findings suggest that hospital rounds conducted by RDs, individualized dietary counseling for inpatients, and the provision of fully personalized meals based on patient preferences are all factors associated with stronger collaboration with the PCT [22,23]. These results highlight the importance of RDs actively participating as integral members of the PCT to ensure that meals are customized according to patients’ conditions and preferences [24,25].

### 4.2. Ward Rounds and Challenges Faced by RDs in Palliative Care

This study underscores the significance of ward rounds as a key skill required for RDs in palliative care. During ward rounds, it is important to monitor food intake and assess various factors such as the patient’s performance status, skeletal muscle mass, levels of anxiety and depression, and degree of fatigue [26]. Incorporating these assessments into nutritional management strategies enables more comprehensive and patient-centered care [27].

RDs frequently encounter difficult situations when dealing with palliative care patients (Figure 1). Many respondents expressed an awareness of their own limitations or were troubled by their inability to provide adequate responses. Our findings suggest that reducing challenging situations for RDs in palliative care may be facilitated by providing pre-graduate training, obtaining specialized certifications in cancer care, and establishing a system that ensures meals are provided in accordance with patients’ preferences. However, participation in meal rounds and post-graduate training were associated with an increase in challenging situations. This may be due to the increased accessibility of dietitians to patients and their families, leading to a greater awareness of the complex roles and responsibilities required in palliative care [28,29]. As the number of RDs assigned to palliative care wards increases and they gain more exposure to the requests and distress of patients and families through ward rounds and nutritional guidance, RDs may experience emotional strain and a loss of confidence [11]. To prevent burnout and foster a sense of professional fulfillment, it is essential to enhance educational opportunities for less experienced and younger RDs [30,31]. Promoting multidisciplinary nutrition support and encouraging a collaborative approach in patient care are crucial steps toward achieving this goal [32,33].

### 4.3. Need for Specialized Training

Previous studies have reported that various factors, such as years of clinical experience, familiarity with practice guidelines, the implementation of multidimensional patient assessments, and educational background, significantly influence an RD’s role in managing cancer cachexia [11]. Moreover, research on interdisciplinary care practices in cachexia management has reported that effective collaboration alleviates physical symptoms, reduces psychological distress, enhances adherence to treatment, and supports both patients and families in adjusting to and coping with cachexia in addition to the RD’s involvement in providing guideline-based nutritional and exercise therapy [16]. Educating patients and families about cancer cachexia, as well as participating in end-of-life discussions as part of a multidisciplinary team, has been shown to strengthen RD confidence [11,34]. These studies highlight the importance of collaboration with the PCT, comprehensive patient assessments during ward rounds, and active engagement with both patients and their families in implementing comprehensive nutritional support. They are consistent with the recommendations of the present guidelines [5,6,17,18,33]. This study also found that providing psychological support to address eating-related distress is an important responsibility of the RD that requires specialized skills [35,36]. Therefore, it is important to support the training of RDs specializing in palliative care to enhance their ability to contribute effectively to multidisciplinary teams.

### 4.4. Study Limitations

The response rate for the survey was 63.9%, suggesting that the data likely reflect the perspectives of RDs with a strong awareness of research collaboration and the development of palliative care. Accordingly, the findings may not be fully generalizable to all dietitians involved in palliative care. Nevertheless, given that the typical response rate for anonymous mailed surveys in Japan is approximately 30%, the results of this study may indicate a meaningful trend.

Moreover, as the survey was based on the subjective experiences of RDs in Japan, future studies should incorporate international comparisons and utilize objective indicators to enhance the generalizability and reliability of the findings [37]. It is also important to acknowledge that this study did not evaluate the direct effects of the reported interventions on patients and their families. Future research should aim to assess these outcomes to better understand the potential benefits of such interventions.

Ultimately, cancer patients receiving palliative care will significantly benefit from an enhanced quality of care when RDs are effectively trained in the design and management of multidisciplinary teams [6,14]. Moreover, empowering RDs to actively engage in research on nutritional interventions in cancer care is essential to strengthening their capacity to deliver robust, evidence-based nutritional support [38].

## 5. Conclusions

Palliative care is a holistic approach that addresses the physical suffering of cancer patients and their psychological and psychosomatic distress. This study investigated the roles and challenges faced by RDs involved in palliative care and identified the key work responsibilities and collaborative systems that contribute to enhanced cancer patient care. The survey results suggest that RDs involved in palliative care must strengthen their collaboration with the PCT, participate in ward rounds, and engage in both pre- and post-graduate education. Furthermore, RDs often face challenging situations in patient care and recognize their own limitations, which can cause distress when they feel unable to provide an adequate response.

Successfully providing individualized meals tailored to patients’ conditions and preferences, as well as addressing eating-related distress, may contribute to a sense of professional fulfillment for RDs and help prevent burnout. It is essential to offer further educational opportunities for less experienced RDs. Establishing a robust collaborative system and fostering education for RDs to practice as integral members of a multidisciplinary PCT are important for improving care quality and supporting the well-being of both patients and healthcare providers.

## Figures and Tables

**Table 1:** Characteristics of registered dietitians (n = 325).

Characteristics	n (%)
**Age**	
20–29 years old	27 (8.3)
30–39 years old	99 (30.5)
40–49 years old	124 (38.2)
50–59 years old	66 (20.3)
60 years old or over	8 (2.5)
**Sex**	
Male	28 (8.6)
Female	276 (84.9)
**Area of Practice**	
Exclusively engaged in palliative care	60 (18.5)
Exclusively engaged in cancer care	11 (3.4)
Concurrently in another area	254 (78.2)
**Years of Experience in Cancer Care**	
0–5 years	108 (33.2)
6–10 years	107 (32.9)
11–15 years	49 (15.1)
16–20 years	36 (11.1)
21 years or more	11 (3.4)
**Certifications**	
Certification as a registered dietitian specializing in pathophysiology	172 (52.9)
Certification as a registered dietitian specializing in cancer	139 (42.8)
**Duties**	
Nutritional management	309 (95.1)
Nutritional counseling	308 (94.8)
Conferences	290 (89.2)
Meal rounds	185 (56.9)
Menu planning	89 (27.4)
Cooking	9 (2.8)
Catering and serving	15 (4.6)
Ingredient management	21 (6.5)
**Nutritional Counseling Responsibilities**	
Nutritional counseling for palliative care inpatients	229 (70.5)
Nutritional counseling for palliative care outpatients	144 (44.3)
**Number of Ward Rounds**	
Daily	129 (39.7)
Several times a week	157 (48.3)
Several times a month	24 (7.4)
Occasionally in a year	11 (3.4)
**Academic Society Memberships**	
Japan Society of Metabolism and Clinical Nutrition	252 (77.5)
Japanese Society for Parenteral and Enteral Nutrition Therapy	185 (56.9)
The Japanese Society of Nutrition and Dietetics	18 (5.5)
Japanese Society for Palliative Medicine	14 (4.3)
The Japanese Society of Clinical Nutrition	13 (4.0)
Japanese Society on Nutrition Care and Management	7 (2.2)
Japanese Association of Supportive Care in Cancer	3 (0.9)
Japan Society of Clinical Oncology	1 (0.3)
Japan Psycho-Oncology Society	0 (0.0)
**Education in Palliative Care**	
Pre-graduate education	48 (14.8)
Post-graduate education	238 (73.2)

**Table 2:** Characteristics of facilities associated with registered dietitians (n = 325).

Characteristics	n (%)
**Meal Provision System**	
Directly operated by hospital	61 (18.8)
Outside contractors	188 (57.8)
Partially outsourced to outside contractors	72 (22.2)
**Number of Beds in Palliative Care Units**	
0 beds (= Palliative care provided in general wards)	170 (52.3)
1–9 beds	28 (8.6)
10–19 beds	40 (12.3)
20–29 beds	63 (19.4)
≥30 beds	10 (3.1)
**Type of Meal Provision**	
Individualized meals	179 (55.1)
Palliative care meals	79 (24.3)
Standard therapeutic meals	67 (20.6)
**Level of Individualized Meal Provision**	
Fully individualized meals based on patient preferences	61 (18.8)
Partially individualized meals	237 (72.9)
No individualized meals	21 (6.5)
**Level of Collaboration with the Palliative Care Team**	
Consistently collaborated	241 (74.2)
Collaborated as needed	84 (25.8)
**Level of Collaboration with the Nutrition Support Team**	
Consistently collaborated	52 (16.0)
Collaborated as needed	273 (84.0)

**Table 3:** The role of registered dietitians and their collaboration with the palliative care team (n = 325).

Variable	Consistently Collaborated (CC) Group (n = 241)	Collaborated As-Needed (AN) Group (n = 84)	*p*-Value
	n (%)	n (%)	
**Certification**			
Certification as a registered dietitian specializing in pathophysiology	129 (53.5)	43 (51.2)	0.800
Certification as a registered dietitian specializing in cancer	108 (44.8)	31 (36.9)	0.249
**Responsibilities**			
Exclusively engaged in palliative or cancer care	62 (25.7)	(10.7)	0.003 *
Concurrently in other departments	179 (74.3)	75 (89.3)	
**Nutritional Counseling Responsibilities**			
For palliative care inpatients	180 (74.7)	(58.3)	0.005
For palliative care outpatients	114 (47.3)	30 (35.7)	0.066
**Duties**			
Meal rounds	138 (57.3)	47 (56.0)	0.222
**Ward Rounds**			
Daily	102 (42.3)	27 (32.1)	<0.001 *
Several times a week	122 (50.6)	35 (41.7)	
Several times a month	11 (4.6)	13 (15.5)	
Several times a year	3 (1.2)	8 (9.5)	
**Provision of Meals**			
Individualized meals	146 (60.6)	33 (39.3)	0.004 *
Palliative care meals	48 (19.9)	31 (36.9)	
Standard therapeutic meals	47 (19.5)	20 (23.8)	
**Level of Individualized Meal Provision**			
Fully individualized meals based on patient preferences	50 (20.7)	11 (13.1)	0.035 *
Partially individualized meals	174 (72.2)	63 (75.0)	
No individualized meals	12 (5.0)	9 (10.7)	
**Education in Palliative Care**			
Pre-graduate education	37 (15.4)	11 (13.1)	0.241
Post-graduate education	181 (75.1)	57 (67.9)	0.167

*p*-values were calculated using Fisher’s exact test for nominal variables and the Mann–Whitney U test for ordinal variables. * *p* < 0.05.

**Table 4:** Multivariate analysis of factors influencing palliative care team collaboration.

Independent Variable	Odds Ratio (95% CI)	*p*-Value
**Years of experience in cancer care**	1.03 (0.98–1.09)	0.269
**Certification as a registered dietitian specializing in cancer**	1.13 (0.59–2.19)	0.713
**Exclusively engaged in palliative care or cancer care**	3.18 (1.27–7.98)	0.014 *
**Ward rounds** (reference: occasionally in a year)		
Several times a month	2.32 (0.46–11.83)	0.310
Several times per week	9.35 (2.19–39.97)	0.003 *
Daily	9.70 (2.23–42.12)	0.002 *
**Type of meal provision** (reference: standard therapeutic meals)		
Palliative care meals	1.68 (0.60–4.70)	0.322
Individualized meals	3.50 (1.01–12.07)	0.048 *

Dependent variable: the degree of collaboration with the PCT, categorized into two groups, those who consistently collaborated and those who collaborated as needed. Multivariate analysis: logistic regression analysis; CI: confidence interval. * *p* < 0.05.

**Table 5:** Multivariate analysis of factors influencing challenging situations for RDs in palliative cancer care.

Dependent Variable	Independent Variable	Odds Ratio (95% CI)	*p*-Value
I struggled with determining appropriate food choices for patients unable to eat.	Years of experience in cancer care	1.04 (0.92–1.16)	0.555
	Certification as a registered dietitian specializing in cancer	0.30 (0.06–1.53)	0.148
	Exclusively engaged in palliative care or cancer care	2.71 (0.32–22.96)	0.361
	Meal rounds	0.41 (0.08–2.19)	0.299
	Fully individualized meals based on patient preferences	0.22 (0.05–0.88)	0.032 *
	Level of collaboration with the palliative care team	0.32 (0.04–2.54)	0.279
	Pre-graduate education	1.03 (0.11–9.26)	0.982
	Post-graduate education	0.44 (0.05–3.92)	0.462
Metabolic complications, such as cachexia, hindered my ability to provide adequate nutritional support.	Years of experience in cancer care	0.97 (0.89–1.05)	0.466
	Certification as a registered dietitian specializing in cancer	1.90 (0.55–6.60)	0.310
	Exclusively engaged in palliative care or cancer care	3.88 (0.48–31.3)	0.203
	Meal rounds	1.69 (0.59–4.82)	0.327
	Fully individualized meals based on patient preferences	0.30 (0.11–0.85)	0.023 *
	Level of collaboration with the palliative care team	1.87 (0.61–5.69)	0.274
	Pre-graduate education	0.75 (0.19–2.91)	0.674
	Post-graduate education	1.28 (0.40–4.06)	0.681
I was unable to meet patients’ dietary wishes.	Years of experience in cancer care	0.96 (0.89–1.04)	0.322
	Certification as a registered dietitian specializing in cancer	2.05 (0.67–6.23)	0.207
	Exclusively engaged in palliative care or cancer care	0.58 (0.20–1.67)	0.313
	Meal rounds	0.55 (0.20–1.50)	0.240
	Fully individualized meals based on patient preferences	0.35 (0.15–0.86)	0.022 *
	Level of collaboration with the palliative care team	0.90 (0.27–2.98)	0.862
	Pre-graduate education	0.22 (0.08–0.60)	0.003 *
	Post-graduate education	0.86 (0.28–2.65)	0.786
I felt that my overall knowledge and experience in palliative care were insufficient.	Years of experience in cancer care	0.96 (0.90–1.01)	0.095
	Certification as a registered dietitian specializing in cancer	0.39 (0.18–0.84)	0.016 *
	Exclusively engaged in palliative care or cancer care	0.74 (0.32–1.71)	0.481
	Meal rounds	0.47 (0.22–0.98)	0.044 *
	Fully individualized meals based on patient preferences	0.59 (0.29–1.19)	0.140
	Level of collaboration with the palliative care team	1.15 (0.50–2.65)	0.740
	Pre-graduate education	1.61 (0.51–5.08)	0.421
	Post-graduate education	0.79 (0.32–1.96)	0.604
Providing psychological support to the patient was challenging.	Years of experience in cancer care	0.98 (0.93–1.02)	0.308
	Certification as a registered dietitian specializing in cancer	1.54 (0.80–2.97)	0.195
	Exclusively engaged in palliative care or cancer care	1.03 (0.50–2.14)	0.932
	Meal rounds	1.14 (0.65–2.01)	0.645
	Fully individualized meals based on patient preferences	1.15 (0.65–2.02)	0.634
	Level of collaboration with the palliative care team	1.30 (0.70–2.43)	0.409
	Pre-graduate education	1.21 (0.52–2.80)	0.663
	Post-graduate education	1.60 (0.86–2.98)	0.137
The patient refused to eat.	Years of experience in cancer care	0.95 (0.91–1.00)	0.037 *
	Certification as a registered dietitian specializing in cancer	0.85 (0.44–1.64)	0.632
	Exclusively engaged in palliative care or cancer care	1.12 (0.52–2.37)	0.778
	Meal rounds	0.83 (0.46–1.50)	0.535
	Fully individualized meals based on patient preferences	0.82 (0.46–1.46)	0.496
	Level of collaboration with the palliative care team	1.03 (0.52–2.02)	0.932
	Pre-graduate education	0.46 (0.22–0.99)	0.048 *
	Post-graduate education	1.83 (0.95–3.51)	0.071
Providing support to patients’ families was challenging.	Years of experience in cancer care	1.00 (0.96–1.04)	0.992
	Certification as a registered dietitian specializing in cancer	1.18 (0.68–2.03)	0.556
	Exclusively engaged in palliative care or cancer care	1.56 (0.84–2.90)	0.161
	Meal rounds	1.36 (0.84–2.20)	0.214
	Fully individualized meals based on patient preferences	0.67 (0.41–1.10)	0.111
	Level of collaboration with the palliative care team	1.27 (0.73–2.21)	0.399
	Pre-graduate education	1.31 (0.65–2.62)	0.446
	Post-graduate education	2.01 (1.15–3.52)	0.015 *
I was consulted on decisions regarding the continuation or discontinuation of tube feeding, gastrostomy, or intravenous nutrition.	Years of experience in cancer care	1.01 (0.97–1.06)	0.604
	Certification as a registered dietitian specializing in cancer	1.05 (0.61–1.82)	0.862
	Exclusively engaged in palliative care or cancer care	0.89 (0.48–1.66)	0.723
	Meal rounds	2.30 (1.40–3.79)	0.001 *
	Fully individualized meals based on patient preferences	1.34 (0.82–2.20)	0.244
	Level of collaboration with the palliative care team	0.84 (0.47–1.47)	0.533
	Pre-graduate education	1.02 (0.51–2.05)	0.949
	Post-graduate education	1.61 (0.90–2.89)	0.110
I was concerned that eating might cause severe complications such as vomiting or choking.	Years of experience in cancer care	1.00 (0.96–1.05)	0.928
	Certification as a registered dietitian specializing in cancer	0.94 (0.55–1.59)	0.805
	Exclusively engaged in palliative care or cancer care	0.94 (0.52–1.70)	0.832
	Meal rounds	1.03 (0.64–1.66)	0.897
	Fully individualized meals based on patient preferences	1.02 (0.64–1.65)	0.925
	Level of collaboration with the palliative care team	1.21 (0.70–2.10)	0.485
	Pre-graduate education	0.99 (0.51–1.94)	0.978
	Post-graduate education	1.61 (0.93–2.81)	0.090
Differences in opinions among staff members arose due to varying perspectives on patient care.	Years of experience in cancer care	1.03 (0.98–1.08)	0.219
	Certification as a registered dietitian specializing in cancer	1.14 (0.64–2.05)	0.656
	Exclusively engaged in palliative care or cancer care	1.79 (0.96–3.35)	0.068
	Meal rounds	1.06 (0.62–1.80)	0.828
	Fully individualized meals based on patient preferences	0.90 (0.53–1.54)	0.701
	Level of collaboration with the palliative care team	1.33 (0.70–2.52)	0.379
	Pre-graduate education	0.87 (0.40–1.90)	0.724
	Post-graduate education	0.85 (0.46–1.57)	0.596

Dependent variable: Challenging situations for RDs, categorized into two groups, “often/sometimes” vs. “not often/never”. Multivariate analysis: logistic regression analysis. A higher odds ratio indicates a greater number of challenging situations, while a lower odds ratio indicates fewer challenging situations. CI: confidence interval. * *p* < 0.05.

## Data Availability

The original contributions presented in this study are included in the article. Further inquiries can be directed to the corresponding author.

## Abbreviations

RD	Registered dietitian
PCT	Palliative care team
CC group	Consistently collaborated group
AN group	Collaborated as-needed group
